# 

**Published:** 1882-08

**Authors:** 


					﻿Vol. II.
-AUGUST, 1882.
No: 8.
JPHE
HOMEOPATHIC PHYSICIAN,
A MONTHLY JOURNAL OF MEDICAL SCIENCE.
“ yfuugna est veritas, el praevalebit.”
ZEj. CT. LEE, im:. d.
EDITOR.
CONTENTS:
(See inside of Cover.) '
PHILADELPHIA:
No. 2109 CHESTNUT STREET.
,T. O IT 13 O 3ST-
ALFRED HEATH & CQ„ Sole Agents for Great Britain,
114 Ebury Street, Eaton Square. .
Yearly Subscrijttioti, $2.00. invariably in advance.
Entered at the Philadelphia Post-Office, as Second Class Mail Matter.
				

